# Economic Evaluation of an Alternative Drug to Sulfadoxine-Pyrimethamine as Intermittent Preventive Treatment of Malaria in Pregnancy

**DOI:** 10.1371/journal.pone.0125072

**Published:** 2015-04-27

**Authors:** Elisa Sicuri, Silke Fernandes, Eusebio Macete, Raquel González, Ghyslain Mombo-Ngoma, Achille Massougbodgi, Salim Abdulla, August Kuwawenaruwa, Abraham Katana, Meghna Desai, Michel Cot, Michael Ramharter, Peter Kremsner, Laurence Slustker, John Aponte, Kara Hanson, Clara Menéndez

**Affiliations:** 1 ISGlobal, Barcelona Ctr. Int. Health Res. (CRESIB), Hospital Clínic—Universitat de Barcelona, Barcelona, Spain; 2 London School of Hygiene and Tropical Medicine, London, United Kingdom; 3 Manhiça Health Research Center (CISM), Manhiça, Mozambique; 4 Centre de Recherches Médicales de Lambaréné (CERMEL), Albert Schweitzer Hospital, Lambaréné, Gabon; 5 Institute of Tropical Medicine, University of Tübingen, Tübingen, Germany; 6 Faculté des Sciences de la Santé (FSS), Université d’Abomey Calavi, Cotonou, Benin; 7 Ifakara Health Institute, Dar Es Salaam, Tanzania; 8 Kenya Medical Research Institute (KEMRI)/Center for Global Health Research, Kisumu, Kenya; 9 Division of Parasitic Diseases and Malaria, Center for Global Health, Centers for Disease Control and Prevention, Atlanta, USA and Kisumu, Kenya; 10 Institut de Recherche pour le Développement (IRD), Paris, France; 11 Université René Descartes, Paris, France; 12 Department of Medicine I, Division of Infectious Diseases and Tropical Medicine, Medical University of Vienna, Vienna, Austria; Glaxo Smith Kline, DENMARK

## Abstract

**Background:**

Intermittent preventive treatment in pregnancy (IPTp) with sulfadoxine-pyrimethamine (SP) is recommended in HIV-negative women to avert malaria, while this relies on cotrimoxazole prophylaxis (CTXp) in HIV-positive women. Alternative antimalarials are required in areas where parasite resistance to antifolate drugs is high. The cost-effectiveness of IPTp with alternative drugs is needed to inform policy.

**Methods:**

The cost-effectiveness of 2-dose IPTp-mefloquine (MQ) was compared with IPTp-SP in HIV-negative women (Benin, Gabon, Mozambique and Tanzania). In HIV-positive women the cost-effectiveness of 3-dose IPTp-MQ added to CTXp was compared with CTXp alone (Kenya, Mozambique and Tanzania). The outcomes used were maternal clinical malaria, anaemia at delivery and non-obstetric hospital admissions. The poor tolerability to MQ was included as the value of women’s loss of working days. Incremental cost-effectiveness ratios (ICERs) were calculated and threshold analysis undertaken.

**Results:**

For HIV-negative women, the ICER for IPTp-MQ versus IPTp-SP was 136.30 US$ (2012 US$) (95%CI 131.41; 141.18) per disability-adjusted life-year (DALY) averted, or 237.78 US$ (95%CI 230.99; 244.57), depending on whether estimates from Gabon were included or not. For HIV-positive women, the ICER per DALY averted for IPTp-MQ added to CTXp, versus CTXp alone was 6.96 US$ (95%CI 4.22; 9.70). In HIV-negative women, moderate shifts of variables such as malaria incidence, drug cost, and IPTp efficacy increased the ICERs above the cost-effectiveness threshold. In HIV-positive women the intervention remained cost-effective for a substantial (up to 21 times) increase in cost per tablet.

**Conclusions:**

Addition of IPTp with an effective antimalarial to CTXp was very cost-effective in HIV-positive women. IPTp with an efficacious antimalarial was more cost-effective than IPTp-SP in HIV-negative women. However, the poor tolerability of MQ does not favour its use as IPTp. Regardless of HIV status, prevention of malaria in pregnancy with a highly efficacious, well tolerated antimalarial would be cost-effective despite its high price.

**Trials Registration:**

ClinicalTrials.gov NCT 00811421; Pan African Trials Registry PACTR2010020001429343 and PACTR2010020001813440

## Introduction

The negative effects of malaria on economic growth and development in endemic countries are well recognised [1, 2]. During pregnancy, malaria infection may have an adverse economic impact through its harmful effects on maternal and infant health, which increase health system as well as household costs, mainly due to clinical management of cases and to the long term consequences of low birth weight [3–8]. The magnitude of this economic impact in the African region, where nearly 30 million pregnancies per year occur in areas of stable transmission of *Plasmodium falciparum*, is likely to be significant [9].

To prevent malaria in pregnancy in Africa in areas with moderate to high transmission of *P*. *falciparum*, the World Health Organization (WHO) recommends intermittent preventive treatment (IPTp) with sulfadoxine-pyrimethamine (SP) and long lasting insecticide treated nets (LLINs) [10]. In Africa, daily cotrimoxazole prophylaxis (CTXp) is recommended to avert opportunistic infections in human immunodeficiency virus (HIV) positive pregnant women [11]. However, the concomitant use of CTXp and SP is contraindicated due to the increased risk of severe cutaneous reactions [12, 13].

The spread of parasite resistance to SP has raised concerns about the medium and long-term use of this drug for IPTp particularly in south-eastern Africa [14, 15]. HIV adds complexity to malaria prevention strategies as it increases the susceptibility to *P*. *falciparum* infection and reduces the efficacy and effectiveness of antimalarial interventions [16]. Although CTXp has been shown to protect against malaria infection in children and non-pregnant adults [17, 18], there is limited evidence of its efficacy in pregnant HIV-positive women [19]. For these reasons, the evaluation of alternative antimalarials to SP for IPTp as well as strategies to prevent malaria in the particularly vulnerable group of HIV-positive pregnant women has become a public health priority.

To help find alternatives to SP for HIV-negative women and to improve malaria prevention in HIV-positive pregnant women, two multicentre clinical trials were designed to evaluate the safety and efficacy of mefloquine (MQ) as IPTp. The trial in HIV-negative women conducted in Benin, Gabon, Mozambique and Tanzania compared IPTp with MQ (15 mg/kg) *versus* SP [20]. The trial in HIV-positive women conducted in Kenya, Mozambique and Tanzania compared IPTp with MQ (15 mg/kg) *versus* placebo in women receiving daily CTXp [21]. In both trials all women received a long lasting insecticide treated net (LLIN) as part of the study interventions.

The findings from these studies showed the health benefits associated with MQ administration at the dosage of 15 mg/kg. However, tolerability and safety issues will limit the usefulness of MQ, at that dosage, as IPTp [22, 23]. In brief, mainly dizziness and vomiting were significantly more frequent in the MQ groups of both trials and, unexpectedly, in the trial in HIV-positive women, HIV viral load at delivery was found to be higher and the rate of mother to child transmission (MTCT) of HIV was increased in MQ recipients in an exploratory analysis. Despite these concerns, the results from these trials served to provide inputs to model the cost-effectiveness of using an alternative antimalarial drug as IPTp. The resulting economic evaluation has estimated: (i) the incremental cost-effectiveness of an alternative antimalarial to SP as IPTp for HIV-negative pregnant women; (ii) the incremental cost-effectiveness of IPTp with an antimalarial compared to IPTp-placebo for HIV-positive pregnant women taking daily CTXp; (iii) the threshold values of several variables, including drug efficacy and cost, beyond which IPTp with an alternative antimalarial stops being cost-effective.

## Methods

### Ethics statement

The clinical trial study protocol and informed consent forms were reviewed and approved by the Ethics Committees from the Hospital Clínic of Barcelona (Spain), the US CDC and the local regulatory authorities and National Ethics Review Committees from Kenya, Mozambique and Tanzania the Comité Consultatif de Déontologie et d’Ethique (CCDE) from the Institut de Recherche pour le Développement (IRD, in France) and all local regulatory authorities and National Ethics Review Committees from each endemic country participating in the study. The trials were conducted under the provisions of the Declaration of Helsinki and in accordance with Good Clinical Practices guidelines set up by the WHO and by the International Conference on Harmonization. The Ethics Committees waived the requirement for consent for additional ancillary analyses of trials data (including the cost effectiveness study). All participants provided written informed consent for participation in the trials. The cost-effectiveness study was designed, with its own separate protocol, in parallel to the development of the protocol of the two clinical trials. The study was not based on individual level data from the trial participants and it was, thus, independent from the trial protocol. The cost-effectiveness study protocol was approved by the Ethics Committee of the London School of Hygiene and Tropical Medicine; by the Institutional Review Boards of the Ifakara Health Institute (Tanzania) and of the Manhiça Health Research Centre (Mozambique); and by the National Institute for Medical Research in Tanzania.

### Study areas and population

The trials were conducted between 2009 and 2013 in five sub-Saharan African countries: Benin (Allada, Sékou, and Attogon), Gabon (Lamberéné and Fougamou), Kenya (Siaya), Tanzania (Makole and Chamwino) and Mozambique (Manhiça and Maragra). All sites were located either in rural or in semi-rural areas with different malaria endemicities (S1 Table). The Gross Domestic Product (GDP) per capita for the year 2012 of Benin, Kenya, Mozambique and Tanzania, ranged from a lowest value of US$ 565 (Mozambique) to a highest value of US$ 943 (Kenya). Gabon had the highest GDP per capita (US$ 11,257) [24].

### Study design

The study in HIV-negative women was an open label randomized trial comparing 2-doses of MQ (Lariam) as IPTp *versus* SP (Malastop). The study in HIV-positive women was a double blind placebo controlled trial comparing 3-doses of MQ as IPTp versus placebo in women receiving daily cotrimoxazole (Septrin) prophylaxis. All women received a LLIN (PermaNet) [20, 21].

**Fig 1:**
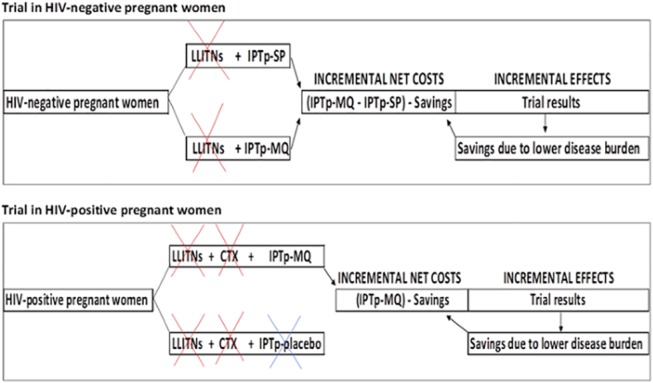
Design of the Incremental economic evaluation. HIV = Human Immunodeficiency Virus; LLITNs = Long-Lasting Insecticide Treated Nets; CTX = Cotrimoxazole; IPTp = Intermittent Preventive Treatment of malaria in pregnancy; SP = Sulphadoxine-Pyrimethamine; MQ = Mefloquine. The figure shows that for both Trials the cost of LLITNs was not considered in the economic evaluation performed as women in both arms were administered with this preventative tool. The same applies for CTX in Trial on HIV-positive women. IPTp-placebo in Trial on HIV-positive women was considered as “doing nothing” option with no costs associated. Incremental net costs were calculated, which included the incremental costs of the intervention minus treatment savings due to its efficacy.

The economic evaluation of the two trials was based on incremental costs, net of health system treatment cost savings, and health outcomes (Fig 1). Although they investigate the cost-effectiveness of the same strategy, namely IPTp-MQ, the two models are separate and independent as they referred to different cohorts (HIV-negative and HIV-positive pregnant women) and have different comparator strategies (IPTp-SP and IPTp-placebo). The outcomes of the two models will be, thus, not comparable. As clinical trials were not designed to make across site comparisons, results of the cost-effectiveness analyses will not be either reported or compared across country [20, 21].

### Study outcomes

The study outcomes used in this economic evaluation were derived from the intention to treat analyses of the trials and were selected according to two pre-defined criteria, namely having a direct health impact [25–27], and showing a statistically significant difference between the intervention groups [28]. All trials outcomes are reported in S2–S5 Tables. In the trial in HIV-negative women, the primary endpoint (prevalence of low birth weight babies) met the first criterion but not the second one since no significant difference was found between MQ and SP groups (Risk Ratio 1.02 (95%CI 0.86; 1.22); p = 0.801). In the trial in HIV-positive women, the primary endpoint (prevalence of peripheral maternal malaria infection at delivery) was significantly lower in women receiving IPTp-MQ compared to those receiving IPTp-placebo (Risk Ratio 0.47 (95%CI 0.27; 0.82); p = 0.008). However, this was an intermediate outcome with lower direct public health relevance compared to other trial outcomes such as morbidity [27]. Accordingly, the outcomes included were the incidence of clinical malaria and maternal anaemia at delivery (Hb<11 g/dl)(both lower in the MQ group compared with the SP group (Table 1)) in the trial in HIV-negative women, and the incidence of non-obstetric hospital admissions (lower in the MQ group compared with the placebo group (Table 2)) in the trial in HIV-positive women. These health outcomes were translated into disability adjusted life years (DALYs) using the parameters shown in Tables 1 and 2.

**Table 1:** Input variables of the threshold and cost-effectiveness analyses of IPTp-MQ compared with IPTp-SP in HIV-negative pregnant women.

	Values (range/CI95%)	Distribution	Estimated parameters	Source
**Costs estimate**				
Time nurses take to provide 1 dose Sulfadoxine-Pyrimethamine (SP); minutes	2.5	Triangular symmetric	Min = 2; Max = 3	Self reported by nurses
Time nurses take to provide 1 dose Mefloquine (MQ); minutes	5.5	Triangular symmetric	Min = 5; Max = 6	Self reported by nurses
Nurses' monthly cost of labour; average including Gabon (US$)	413.25	Gamma	α = 11.52; β = 35.87	Country MoH
Nurses' monthly cost of labour; average excluding Gabon (US$)	336.73	Gamma	α = 283.47; β = 1.19	Country MoH
MQ (250mg) price per tablet (US$)	0.5	Triangular asymmetric	Min = 0.46; Max = 0.90	(MMV + GF)*
International SP (500 mg + 25 mg) price per tablet (US$)	0.07	Lognormal	μ = -2.66; σ = 0.04	GF**
Average number of tablets per dose MQ—includes drug wastage	3.75	Point estimate	-	[20, 21]
Average number of tablets per dose SP—includes drug wastage	3.15	Point estimate	-	Trial data
**Disability-Adjusted Life Years (DALYs) estimate (not age-weighted)**				
Discount rate	0.03	Point estimate	-	Assumption
Average women's age (years)	24.5	Point estimate		[20]
Length Disability—malaria (years)	0.01(0.005–0.02)	Gamma	α = 9.89; β = 0.001	Experts opinion
Length Disability—anaemia malaria related (years)	0.06 (0.04–0.11)	Gamma	α = 8.31; β = 0.007	[71]
Across country average life expectancy of women aged 24.5 years (in years, for the year 2011)—including Gabon	46.51	Point estimate	-	[72]
Across country average life expectancy of women aged 24.5 years (in years, for the year 2011)—excluding Gabon	45.63	Point estimate	-	[72]
Sensitivity analysis: life expectancy of Japanese women aged 24.5 (in years, for the year 2011)	66.28	Point estimate	-	[72]
Disability weight (moderate anaemia)	0.058 (0.04–0.09)	Lognormal	μ = -2.85; σ = 0.21	[73]
Disability weight (infectious disease: acute episode, severe)	0.21 (0.14–0.30)	Lognormal	μ = -1.56; σ = 0.19	[73]
**DALYs averted estimate**				
Incidence of clinical malaria SP arm (episodes per PYAR)	0.17	Point estimate	-	[20]
Clinical malaria Relative Risk MQ arm—First or only episode. [Incidence (Episodes person/year) in the SP group = 0.17; incidence in the MQ group = 0.12]	0.67 (0.52–0.88)	Lognormal	μ = -0.42; σ = 0.14	[20]
Anaemia at delivery (Hb<11 g/dl) Relative Risk MQ arm [Prevalence in the SP group = 44.1; prevalence in the MQ group = 40.5]	0.92 (0.85–0.99)	Lognormal	μ = -0.08; σ = 0.04	[20]
Case fatality ratio malaria %	0.33 (0.26–0.45)	Beta	α = 3.91; β = 1182.49	[29]
Case fatality ratio anaemia %	1	Beta	α = 98.99; β = 9800.01	[30]
**Adherence to intervention**				
Drop out rate first dose MQ %	2	Beta	α = 97.98; β = 4801.02	[20]
Drop out rate second dose MQ %	14	Beta	α = 85.86; β = 527.43	[20]
Drop out rate first dose SP %	1	Beta	α = 98.99; β = 9800.01	[20]
Drop out rate second dose SP %	8	Beta	α = 91.92; β = 1057.08	[20]
**Savings due to the efficacy of IPTp-MQ compared with IPTp-SP**				
% of malaria cases seeking care	50	Triangular symmetric	Min = 40; Max = 60	[29]
% of malaria cases in pregnancy that are severe	6	Point estimate	-	[20]
*With the inclusion of Gabon*:				
Health system inpatient savings per malaria episode (US$)	188.68	Gamma	α = 2.14; β = 88.06	Our estimate
Health system outpatient savings per malaria episode (US$)	11.61	Gamma	α = 5.01; β = 2.31	Our estimate
Households’ outpatient direct treatment savings per malaria episode (US$)	13.47	Gamma	α = 0.14; β = 93.39	Our estimate
Households’ outpatient indirect treatment savings per malaria episode (US$)	1.58	Gamma	α = 0.16; β = 9.62	Our estimate
Households’ inpatient direct treatment savings per malaria episode (US$)	89.15	Gamma	α = 0.43; β = 204.60	Our estimate
Households’ inpatient indirect treatment savings per malaria episode (US$)	2.69	Gamma	α = 0.12; β = 22.07	Our estimate
*With the exclusion of Gabon*:				
Health system inpatient savings per malaria episode (US$)	40.65	Gamma	α = 100; β = 0.41	Our estimate
Health system outpatient savings per malaria episode (US$)	4.32	Gamma	α = 100; β = 0.04	Our estimate
Households’ outpatient direct treatment savings per malaria episode (US$)	2.16	Gamma	α = 0.16; β = 13.74	Our estimate
Households’ outpatient indirect treatment savings per malaria episode (US$)	0.8	Gamma	α = 0.17; β = 4.65	Our estimate
Households’ inpatient direct treatment savings per malaria episode (US$)	20.97	Gamma	α = 0.56; β = 37.32	Our estimate
Households’ inpatient indirect treatment savings per malaria episode (US$)	1.75	Gamma	α = 0.13; β = 13.63	Our estimate

All monetary values are expressed in US$ 2012

IPTp = Intermittent Preventive Treatment of malaria in pregnancy; HIV = Human Immunodeficiency Virus

MQ = Mefloquine; SP = Sulphadoxine-Phyrimethamine; PYAR = Person-year at risk; RR = Relative Risk.

α, β are the parameters of the Beta and Gamma distributions and μ, σ are the parameters of the lognormal distribution.

*Medicine for malaria venture price under negotiation and ranges given by lowest and highest Global Fund procurement prices.

**The average Global Fund procurement price for SP in Sub-Saharan Africa for the year 2012.

**Table 2:** Input variables of the threshold and cost-effectiveness analyses of IPTp-MQ compared with IPTp-placebo in HIV-positive pregnant women.

	Values (range/CI 95%)	Distribution	Estimated Parameters	Source
**Costs estimate**				
Time nurses take to provide 1 dose MQ (minutes)	5.5	Triangular symmetric	Min = 5; Max = 6	Self reported by nurses
Nurses' monthly cost of labour (US$)	332.17	Gamma	α = 205.95; β = 1.61	Country MoH
MQ (250 mg) price per tablet (US$)	0.51	Triangular asymmetric	Min = 0.46; Max = 0.9	MMV + GF*
Average number of tablets per dose MQ—includes drug wastage	3.75	Point estimate	-	[21]
**Disability-Adjusted Life Years (DALYs) estimate**				
Discount rate	0.03	Point estimate	-	Assumption
Incidence of all cause admissions placebo arm (episodes per PYAR)	0.35	Point estimate	-	[21]
Non-obstetric hospital admissions Relative Risk MQ arm [Incidence (episodes per person/year) placebo group = 0.35; incidence MQ group = 0.20]	0.59 (0.37–0.95)	Lognormal	μ = -0.49; σ = 0.24	[21]
Across country average Life Expectancy of women aged 26.5 years (in years, for the year 2011)	44.06	Point estimate	-	[72]
Sensitivity analysis: Life Expectancy of Japanese women aged 26.5 (in years, for the year 2011)	61.38	Point estimate	-	[72]
Disability weights infectious diseases (acute severe episode)	0.21 (0.14–0.29)	Lognormal	μ = -1.56; σ = 0.19	[73]
Disability weights moderate abdominopelvic problem	0.12 (0.08–0.18)	Lognormal	μ = -2.09; σ = 0.19	[73]
Disability weights severe anaemia	0.164 (0.11–0.23)	Lognormal	μ = -1.81; σ = 0.18	[73]
Length disability	0.0273	Gamma	μ = 100; σ = 0.0003	
**DALYs averted estimate**				
Case fatality ratio HIV positive adults with all cause co-morbidity %	35	Beta	α = 64.65; β = 120.06	[31]
Case fatality ratio severe anaemia %	1.4	Beta	α = 1.03; β = 72.60	[30]
Case fatality ratio maternal disorders %	3.4	Beta	α = 96.57; β = 2,743.61	[32]
**Adherence to intervention**				
Drop out rate first dose MQ %	2	Beta	α = 97.98; β = 4,801.02	[21]
Drop out rate second dose MQ %	10	Beta	α = 89.9; β = 809.10	[21]
Drop out rate third dose MQ %	19	Beta	α = 80.81; β = 344.50	[21]
**Savings due to the efficacy of IPTp-MQ compared with IPTp-SP**				
*Health system savings* (US$)				
Health system inpatient savings per hospitalisation episode (US$)	28.48	Gamma	α = 6.48; β = 4.39	Our estimate
*Households’ inpatient treatment savings* (US$)				
Direct	10.92	Gamma	α = 6.48; β = 4.39	Our estimate
Indirect	5.34	Gamma	α = 6.48; β = 4.39	Our estimate

All monetary values are expressed in US$ 2012

IPTp = Intermittent Preventive Treatment of malaria in pregnancy; HIV = Human Immunodeficiency Virus

MQ = Mefloquine; SP = Sulphadoxine-Phyrimethamine; PYAR = Person-year at risk; RR = Relative Risk.

α, β are the parameters of the Beta and Gamma distributions and μ, σ are the parameters of the lognormal distribution.

*Medicine for malaria venture price under negotiation and ranges given by lowest and highest Global Fund procurement prices.

Dizziness and vomiting were the most frequent tolerability adverse events related to medication being significantly higher in the MQ groups of both trials. Approximately 35% of the study women experienced either transient dizziness or vomiting in the MQ groups compared with 10% in the comparator groups. However, only about 25% of these adverse events were moderate and less than 5% severe and likely to interfere with normal daily life activities. Low tolerability was translated into household indirect costs.

### Disability adjusted life years

For both trials, the number of episodes averted for a target of 1000 women receiving IPTp was defined as the protective efficacy (1-RR) times the incidence of the episodes (person-year at risk) in the comparator group, times 1000, in the two trials. The number of deaths averted was obtained by multiplying the number of episodes averted by the case fatality ratio, specific to pregnant women where data were available [29–32]. DALYs averted were calculated by multiplying the number of DALYs due to the disease by the reduction in morbidity and mortality resulting from the intervention. DALYs were discounted but not age-weighted [33]. For the trial in HIV-negative women, total DALYs averted were the sum of DALYs averted for clinical malaria and for anaemia. For the trial in HIV-positive women, total DALYs averted were the sum of DALYs averted for each of the three categories of non-obstetric hospital admission (moderate abdominopelvic problems; infectious diseases (acute severe episode); and severe anaemia (Hb<7 g/dl)) (Table 2).

### Cost savings

Health system and household cost savings were calculated by multiplying inpatient and outpatient treatment costs by the number of admissions and outpatient episodes averted, respectively. For the trial in HIV-negative pregnant women, both health system and household costs for an outpatient visit and for an admission due to malaria were estimated based on data from Tanzania and Mozambique [29]. In Tanzania, an *ad hoc* exit survey was undertaken among 225 pregnant or post-postpartum women with malaria (71 were inpatients and 154 outpatients) [34]. Health system costs were extrapolated to the remaining countries based on the ratios between WHO-Choice outpatient and inpatient costs of Benin and Gabon and the average previously estimated costs of Mozambique and Tanzania [35]. For example, health system costs of Benin were estimated by multiplying the average health system costs of Mozambique and Tanzania by the ratio between the average WHO-Choice costs of Mozambique and Tanzania and WHO-Choice costs of Benin. Using the same approach, the average household costs from Mozambique and Tanzania for malaria treatment were extrapolated to Benin and Gabon based on the estimates of a previous study [36]. Inpatient malaria episodes averted were the total number of malaria episodes averted times the proportion of malaria cases hospitalized [37]. Outpatient malaria episodes averted were the result of the total number of malaria episodes averted times the percentage of presumptive malaria in pregnancy cases seeking care at public health facilities [29]. It was assumed that anaemia was not the cause of either outpatient visits or hospital admissions [38]. Therefore, no treatment savings were associated with this outcome.

In the trial in HIV-positive pregnant women, savings were based on the number of non-obstetric admissions averted in the three categories. Health system admission costs averted in Mozambique and Tanzania were represented by the malaria treatment figures used for the trial in HIV-negative pregnant women, while estimates from a published study were used for Kenya [39]. Admission costs averted for the households were the same as for the trial in HIV-negative pregnant women in the case of Mozambique and Tanzania [29, 34] and estimated from a cross-sectional study recently conducted among pregnant women with malaria in the case of Kenya (Hill, J and Webster, J, unpublished data).

For both trials, household cost savings were split into direct and indirect. Direct savings referred to medical (e.g. drugs, diagnostics) and non-medical (e.g. transportation) costs averted due to fewer malaria episodes and non-obstetric admissions. Indirect cost savings had two components, the aversion of time lost due to malaria and to non-obstetric admissions and the additional time lost (one day on average) due to poor MQ tolerability. Time was valued at the national minimum salary in force, averaged across countries [40]. All costs were expressed in US$ 2012 and discounted at 3%.

### Intervention costs

Costs associated with IPTp-MQ or with IPTp-SP were estimated as the drug cost plus the value of the health personnel time taken to administer the intervention. Time taken to administer one dose of MQ was reported by the health personnel involved in the trials. Time taken to administer one dose of SP was obtained from a previous study [29]. In the trial in HIV-negative women, incremental costs were calculated as the difference between the time needed to provide MQ and SP multiplied by the value of health personnel time plus the difference between unit cost of drugs (price per tablet) multiplied by the number of tablets administered. The value of health personnel time was the average salary across sites. In the trial in HIV-positive women, IPTp-placebo was considered as a “do-nothing” option with no associated costs, thus the incremental costs were health personnel time to provide three doses of MQ multiplied by the value of their time plus the cost of MQ. Mefloquine tablets of 250 mg were administered in both trials at a dosage of 15 mg/kg. Thus, the number of tablets of MQ per dose was calculated based on women’s median weight at the first IPTp administration. The number of tablets of SP was fixed at three per dose (each tablet containing 500 mg sulfadoxine and 25 mg pyrimethamine) [10]. The price of SP was estimated using the average Global Fund procurement price in Sub-Saharan Africa for the year 2012, augmented by 10% to account for insurance and freight, and by an additional 15% to include local transportation costs, leading to a cost of US$ 0.07 per tablet. The average international market price of MQ for the year 2012 (including insurance, shipment and local transportation), was nearly 1 US$ per tablet [41]. Despite being present in the private market of some sub-Saharan African countries for many years [42], widespread use of MQ as mono-therapy has never occurred for either malaria prevention or treatment in Africa. For this reason, there is not a large scale programme price for MQ in the region and for the purpose of this study. Its average price was estimated by doubling the active pharmaceutical ingredient price of 800 US$/kilogram to include formulation and packing costs and by adding 10% for freight and insurance and 15% for local transportation costs [43]. This gave a mean MQ price of US$ 0.50 per tablet. To allow for uncertainty the minimum and maximum Global Fund procurement price of MQ in Asia, where MQ is used in public health programmes, were used as the range [44].

Intervention costs were calculated for 1000 pregnant women receiving IPTp. Trial attrition was considered in cost calculation by reducing the number of doses at each administration resulting from all-cause women’s drop-out. Input values are presented in Table 1 for the trial in HIV-negative pregnant women and in Table 2 for the trial in HIV-positive pregnant women.

### Cost effectiveness analyses

The health system perspective was used for the net incremental cost-effectiveness ratios (ICERs) calculation. ICERs were defined as net as health system cost savings were subtracted from the incremental costs of the intervention. Net ICERs were equal to:

$$\begin{array}{l} \text{Trial in HIV}-\text{negative pregnant women}=\\ \frac{\text{Costs associated with IPTp}-\text{MQ}-\text{Costs associated with IPTp}-\text{SP}\;-\;\text{Health system savings}}{\text{DALYs averted clinical malaria}\;+\;\text{DALYs averted anaemia}}\\ \text{Trial in HIV}-\text{positive pregnant women}=\\ \frac{\text{Costs associated with IPTp}-\text{MQ}-\text{Health system savings}}{\text{DALYs averted non}-\text{obstetric hospital admissions}} \end{array}$$

The maximum level at which IPTp-MQ is cost-effective compared with IPTp-SP (trial in HIV-negative pregnant women) or with the “do-nothing” alternative (trial in HIV-positive pregnant women) was defined by setting a conservative net incremental cost-effectiveness threshold of 240 US$ per DALY averted [45]. This value corresponds to the threshold below which an intervention is considered cost-effective, proposed by the World Bank in 1993 updated to 2012 prices [46].

Being the country with the highest GDP per capita, Gabon also had higher health worker salaries and health system costs compared with the other countries, with a substantial impact on the numerator of the ICER [24]. Cost-effectiveness results were, therefore presented in two scenarios, including and excluding Gabon, respectively.

### Management of uncertainty

All model inputs were expressed as probability distributions (Tables 1 and 2) [47]. Distribution parameters were calculated based on mean values and lower and upper range values. Where lower and upper range values were unknown, a standard deviation of 20% was assumed and distribution parameters were estimated accordingly. Finally, parameters were fitted through maximum likelihood for those variables for which patient or country level data were available, such as household and health system costs. Stata 12.1 (Stata Corporation, College Station, TX, USA) was used to calculate these estimates. Probabilistic sensitivity analysis was performed through Monte Carlo simulations using Microsoft Excel. The cumulative average net monetary benefits (threshold level*incremental effects—incremental costs) were depicted in graphs to evaluate the number of iterations needed to produce stable results. One-way sensitivity analysis was undertaken on life expectancy by changing local values, used in the base scenario, to the life expectancy of Japanese women, globally the longest living. A threshold analysis of the cost-effectiveness of IPTp-MQ was performed to estimate cut-off points beyond which the intervention is no longer cost-effective. Threshold analysis was performed on the MQ price for both trials and on the SP price, malaria incidence, and on protective efficacy against clinical malaria for the trial in HIV-negative pregnant women.

ICERs were presented graphically in the cost-effectiveness plane [48] and as acceptability curves [49, 50]. The cost-effectiveness plane plots all Monte Carlo simulations produced with respect to DALYs averted (X axis) and incremental costs (Y axis), allowing the visualization of the confidence area delimited by the 95% confidence ellipses. Acceptability curves show the probability that IPTp-MQ is cost-effective (Y axis) according to the different levels of policymaker willingness to pay for each DALY averted (X axis).

## Results

In the trial in HIV-negative pregnant women, for 500 women receiving IPTp-MQ in comparison with 500 receiving IPTp-SP, the number of DALYs averted from malaria and anaemia reduction was 9.23 (CI95% 9.09; 9.38) (Table 3). When Gabon was included in the analysis, the incremental cost of delivering two doses of MQ for IPTp was US$ 2,007.21 (CI95% 1,992.48; 2,021.93). Health system savings were US$ 949.10 (CI95% 925.29; 972.91), leading to a net incremental cost of US$ 1,058.11 (CI95% 1,030.16; 1,086.05). The net ICER was US$ 136.30 (CI95% 131.41; 141.18) per DALY averted. Household direct cost savings were US$ 668.97 (CI95% 619.13; 718.81) while indirect cost savings were negative (i.e. were a net cost) estimated at US$ -44.23 (CI95% -49.58; -38.87). When Gabon was excluded, incremental cost was slightly reduced but health system savings were almost four times lower yielding a net ICER of US$ 237.78 (CI95% 230.99; 244.57). Household direct and indirect cost savings decreased by 80% and 15%, respectively, compared with the inclusion of Gabon.

**Table 3:** HIV-negative trial: Results of cost-effectiveness analysis (per 1000 pregnant HIV-negative women).

	Value (CI 95%)
**Health impact of IPTp-MQ on malaria and anaemia**	
Number of maternal clinical malaria episodes averted	55.14 (54.47; 55.81)
Number of maternal anaemia at delivery cases averted	13.39 (13.12; 13.66)
Number of malaria admissions averted	3.31 (3.27; 3.35)
Number of malaria outpatient visits averted	27.14 (27.14; 27.83)
Number of malaria deaths averted	0.18 (0.178; 0.183)
Number of anaemia deaths averted	0.13 (0.131; 0.136)
Number of DALYs averted (malaria + anaemia)	9.23 (9.09; 9.38)
**Cost-effectiveness (including Gabon)**	
Incremental costs (IPTp-MQ—IPTp-SP) (US$)	2,007.21 (1,992.48; 2,021.93)
Health system treatment savings (US$)	949.10 (925.29; 972.91)
Incremental net costs* (US$)	1,058.11 (1,030.16; 1,086.05)
Net incremental costs per DALY averted** (US$)	136.30 (131.41; 141.18)
**Treatment household savings due to IPTp-MQ efficacy on clinical malaria (including Gabon)**	
Households direct treatment savings (US$)	668.97 (619.13; 718.81)
Households indirect treatment savings (US$)	-44.23 (-49.58; -38.87)
**Cost-effectiveness (excluding Gabon)**	
Incremental costs (IPTp-MQ—IPTp-SP) (US$)	1,992.02 (1,977.42; 2,006.63)
Health system treatment savings (US$)	254.61 (251.33; 257.89)
Incremental net costs* (US$)	1,737.41 (1,722.42; 1,752.41)
Net incremental costs per DALY averted** (US$)	237.78 (230.99; 244.57)
**Treatment household savings due to IPTp-MQ efficacy on clinical malaria (excluding Gabon)**	
Households direct treatment savings (US$)	128.29 (120.01; 136.56)
Households indirect treatment savings (US$)	-51.74(-54.49; -48.99)

All monetary values are expressed in US$ 2012

IPTp = Intermittent Preventive Treatment of malaria in pregnancy; HIV = Human Immunodeficiency Virus

MQ = Mefloquine; SP = Sulphadoxine-Phyrimethamine; DALY = Disability-adjusted Life Year.

*Incremental net costs are equal Incremental cost minus Health system treatment savings

**Net incremental costs per DALY averted are equal Incremental net costs divided by number of DALYs averted

In the trial in HIV-positive women, the cost of delivering 3 doses of IPTp-MQ to 1,000 women was US$ 6,667.86 (CI95% 6,625.15; 6,710.56) (Table 4). The number of DALYs averted was 603.33 (CI95% 592.94; 613.72) based on 138.91 (CI95% 136.60; 141.22) non-obstetric admissions averted and on 24.57 (CI95% 24.15; 24.99) deaths averted. Health system savings were nearly US$ 4,000 leading to an incremental net cost of US$ 2,712.96 (CI95% 2,658.30; 2,767.62). The net cost per DALY averted was US$ 6.96 (CI95% 4.22; 9.70). Household direct and indirect savings were US$ 1,519.10 (CI95% 1,470.34; 1,567.86) and US$ 543.90 (CI95% 530.64; 557.16), respectively.

**Table 4:** HIV-positive trial: Results of the cost-effectiveness analysis (per 1000 pregnant HIV-positive women).

	Value (CI 95%)
**Health impact of IPTp-MQ**	
Number of non-obstetric admissions averted	138.91 (136.60; 141.22)
Number of deaths averted	24.57 (24.15; 24.99)
Number of DALYs averted	603.33 (592.94; 613.72)
**Cost-effectiveness**	
Costs associated with IPTp-MQ (US$)	6,667.86 (6.625.15; 6,710.56)
Health system treatment savings (US$)	3,954.90 (3,854.54; 4,055.26)
Incremental net cost* (US$)	2,712.96 (2,658.30; 2,767.62)
Net incremental costs per DALY averted** (US$)	6.96 (4.22; 9.70)
**Treatment savings due to IPTp-MQ efficacy on clinical malaria**	
Households direct savings (US$)	1,519.10 (1,470.34; 1,567.86)
Households indirect savings (US$)	543.90 (530.64; 557.16)

All monetary values are expressed in US$ 2012

IPTp = Intermittent Preventive Treatment of malaria in pregnancy; HIV = Human Immunodeficiency Virus

MQ = Mefloquine; SP = Sulphadoxine-Phyrimethamine; DALY = Disability-adjusted Life Year.

*Incremental net costs are equal to Costs associated with IPTp-MQ minus Health system treatment savings

**Net incremental costs per DALY averted are equal to Incremental net costs divided by Number of DALYs averted

Approximately 1,600 random iterations of the cost-effectiveness model were required to achieve stable results from the two models (with and without Gabon) of the trial in HIV-negative pregnant women S1 Fig Stable results were achieved in the trial in HIV-positive women (S2 Fig) at about 1,800 iterations. With 2,000 iterations the results in the three models can be considered robust.

**Fig 2:**
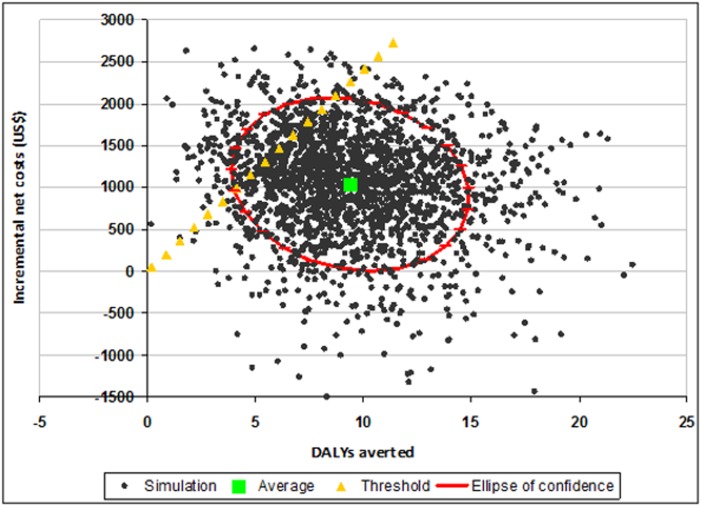
Cost effectiveness plane: IPTp-MQ versus IPTp-SP in HIV-negative pregnant women in Benin, Gabon, Mozambique and Tanzania. IPTp-MQ = Intermittent Preventive Treatment of malaria in pregnancy with Mefloquine; IPTp-SP = Intermittent Preventive Treatment of malaria in pregnancy with Sulphadoxine-Phyrimethamine; HIV = Human Immunodeficiency Virus; DALYs = Disability-adjusted life years. The graph plots 2,000 Monte Carlo simulations of the incremental cost-effectiveness ratio (ICER) with respect to its components: health impact (X-axis) and incremental costs (Y-axis). Average simulation point, ellipse of confidence (95%) and threshold level of the ICER (240 US$/DALY averted) are also depicted. Cost-effective dots are placed below/right the threshold line.

**Fig 3:**
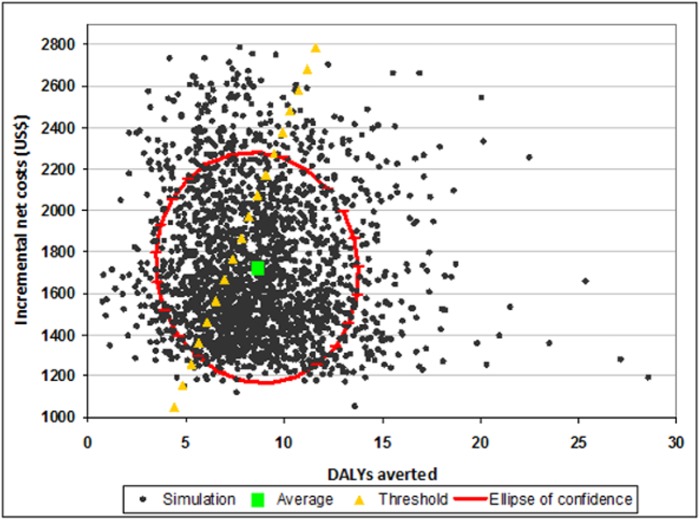
Cost effectiveness plane: IPTp-MQ versus IPTp-SP in HIV-negative pregnant women in Benin, Mozambique and Tanzania. IPTp-MQ = Intermittent Preventive Treatment of malaria in pregnancy with Mefloquine; IPTp-SP = Intermittent Preventive Treatment of malaria in pregnancy with Sulphadoxine-Phyrimethamine; HIV = Human Immunodeficiency Virus; DALYs = Disability-adjusted life years. The graph plots N = 2,000 Monte Carlo simulations of the incremental cost-effectiveness ratio (ICER) with respect to its components: health impact (X-axis) and incremental costs (Y-axis). Average simulation point, ellipse of confidence (95%) and threshold level of the ICER (240 US$/DALY averted) are also depicted. Cost-effective dots are placed below/right the threshold line.

**Fig 4:**
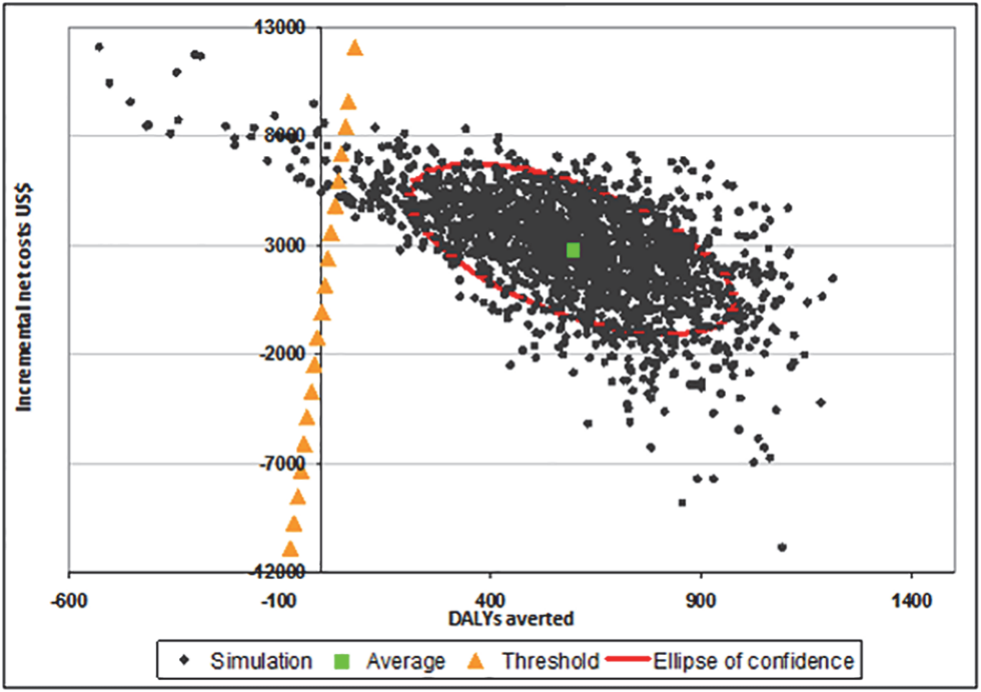
Cost effectiveness plane: IPTp-MQ versus IPTp-Placebo in HIV-positive pregnant women in Kenya, Mozambique and Tanzania. IPTp-MQ = Intermittent Preventive Treatment of malaria in pregnancy with Mefloquine; HIV = Human Immunodeficiency Virus; DALYs = Disability-adjusted life years. The graph plots 2,000 Monte Carlo simulations of the incremental cost-effectiveness ratio (ICER) with respect to its components: health impact (X-axis) and incremental costs (Y-axis). Average simulation point, ellipse of confidence (95%) and threshold level of the ICER (240 US$/DALY averted) are also depicted.

Figs 2–4 show the cost-effectiveness planes. In all the figures the average simulation points are situated below and to the right of the threshold limit indicating that, on average, IPTp-MQ was more cost-effective than the comparators. However, in Fig 2 (trial in HIV-negative women, Gabon included) a fraction of the confidence area lies above and to the left of the threshold limit. Several simulated combinations of incremental costs and health effects exceeded US$ 240 per DALY averted indicating that IPTp-MQ is not cost-effective when the incremental costs are higher than approximately US$ 1,000 and fewer than 8 DALYs are averted. In Fig 3, (trial in HIV-negative women, Gabon excluded) the share of the confidence area lying above/left of the threshold limit is larger, defining a wider non-cost-effective region. For the trial in HIV-positive women the entire confidence area lies below the threshold limit, assessing that the intervention is cost-effective across all possible combinations of intervention costs and health effects (Fig 4).

**Fig 5:**
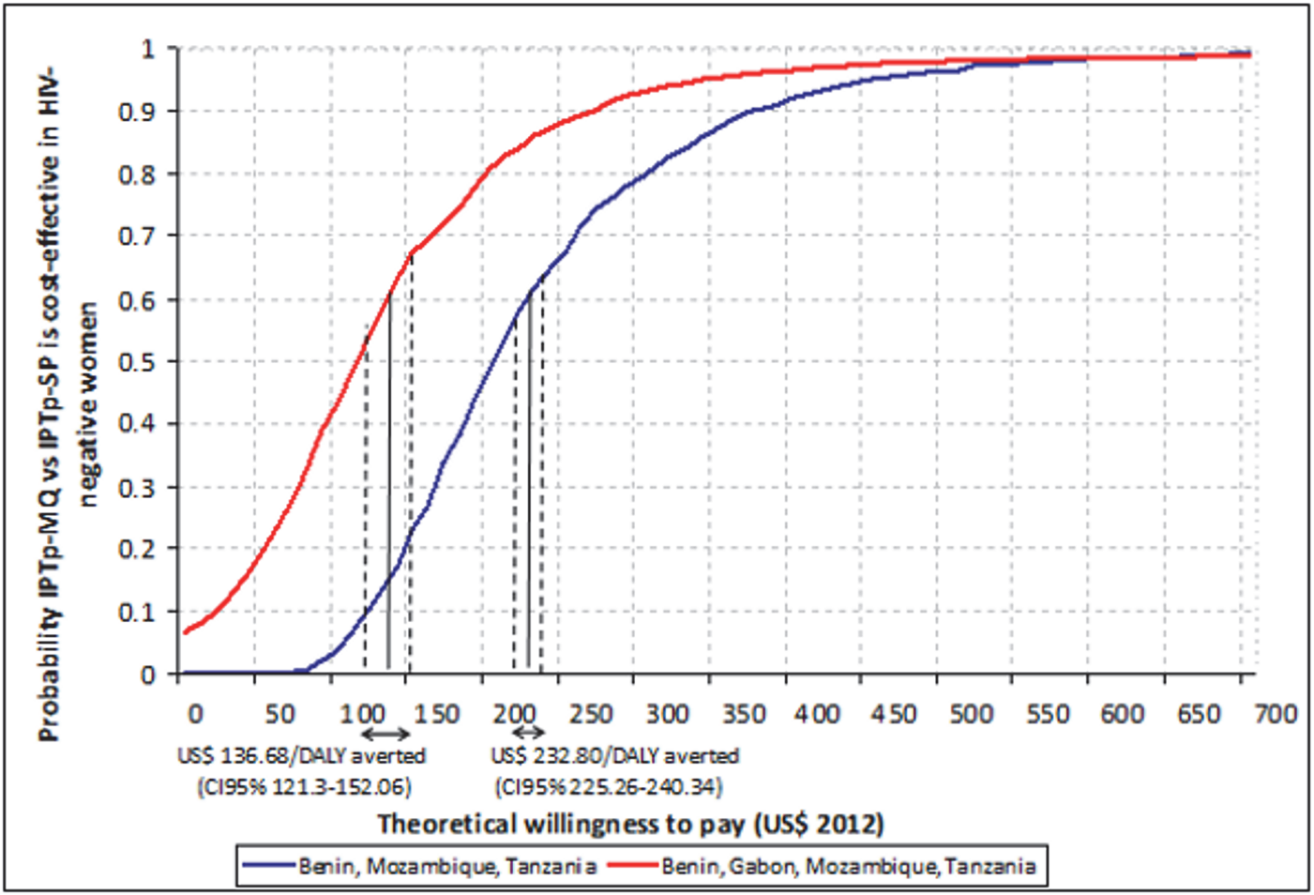
Cost-effectiveness of IPTp-MQ versus IPTp-SP in HIV-negative pregnant women in Benin, Gabon, Mozambique and Tanzania: acceptability curves. IPTp-MQ = Intermittent Preventive Treatment of malaria in pregnancy with Mefloquine; IPTp-SP = Intermittent Preventive Treatment of malaria in pregnancy with Sulphadoxine-Phyrimethamine; HIV = Human Immunodeficiency Virus; DALYs = Disability-adjusted life years. Acceptability curves were constructed by plotting the cumulative distribution of the incremental cost-effectiveness ratios (ICERs) of IPTp-MQ compared with IPTp-SP per DALYs averted. The Y axis is interpreted as probability that the intervention is cost-effective for every level of policy makers’ willingness to pay for each DALY averted (X axis). US$ 136.30 per DALY averted (CI95% 131.41–141.18) is the ICER for the scenario including Benin, Gabon, Mozambique and Tanzania. US$ 237.99 (CI95% 230.99–244.57) per DALY averted is the ICER for the scenario excluding Gabon.

**Fig 6:**
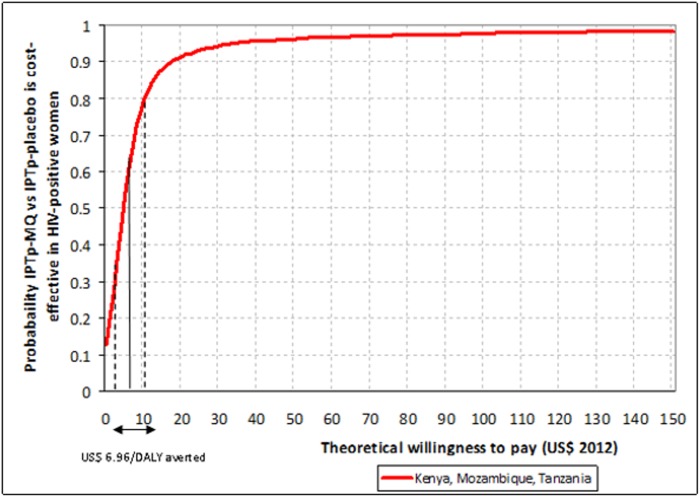
Cost-effectiveness of IPTp-MQ versus IPTp-Placebo in HIV-positive pregnant women in Kenya, Mozambique and Tanzania: acceptability curve. IPTp-MQ = Intermittent Preventive Treatment of malaria in pregnancy with Mefloquine; HIV = Human Immunodeficiency Virus; DALYs = Disability-adjusted life years. The acceptability curve was constructed by plotting the cumulative distribution of the incremental cost-effectiveness ratio (ICER) of IPTp-MQ compared with IPTp-Placebo per DALYs averted. The Y axis is interpreted as probability that the intervention is cost-effective for every level of policy makers’ willingness to pay for each DALY averted (X axis). US$ 6.96 per DALY averted (CI95% 4.22–9.70) is the ICER resulting from the analysis.

The acceptability curves for the trial in HIV-negative women are shown in Fig 5. At each level of theoretical willingness to pay (X-axis) the probability that the intervention is cost-effective (Y-axis) is much higher when Gabon is included in the analysis (red line) than when it is excluded (blue line). To achieve a 90% probability that the intervention is cost-effective policy makers should be willing to pay around 270 US$ per DALY averted (Gabon included) or US$ 380 (Gabon excluded). For the trial in HIV-positive women a willingness to pay of US$ 20 per DALY averted would achieve a 90% probability that the intervention is cost-effective (Fig 6).

Cost-effectiveness results did not change considerably when the life expectancy of Japanese women was used to calculate DALYs averted. For the trial in HIV-negative women, the number of DALYs averted increased to 10.32 (CI95% 10.07; 10.57) and to 9.60 (CI95% 9.38; 9.82) Gabon included or excluded, respectively; while the net ICER decreased to US$ 133.60 (CI95% 117.47; 149.73) and US$ 213.71 (CI95% 202.85; 224.57). For the trial in HIV-positive women, the number of DALYs averted increased to 695.58 (CI95% 676.68; 714.48) and the net ICER fell to US$ 6.26 (CI95% 3.55; 8.97).

**Fig 7:**
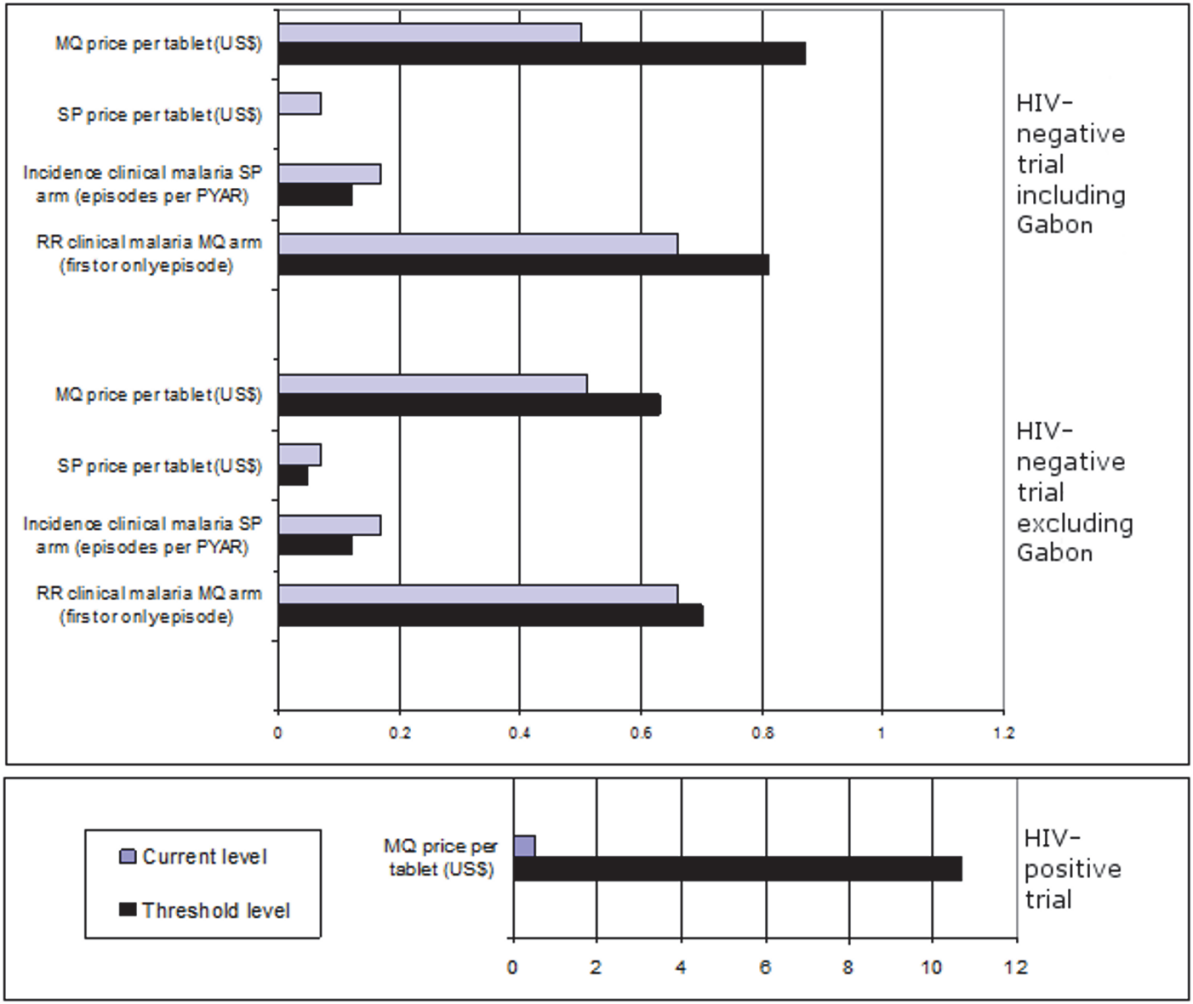
Results of the threshold analysis of IPTp-MQ among HIV-negative and HIV-positive pregnant women. IPTp = Intermittent Preventive Treatment of malaria in pregnancy; HIV = Human Immunodeficiency Virus; MQ = Mefloquine; SP = Sulphadoxine-Phyrimethamine; PYAR = Person-year at risk; RR = Relative Risk. The graph depicts up to which level (threshold level) several variables can increase or decrease from their current level for the interventions to remain cost-effective.

According to the probabilistic threshold analysis (Fig 7) for the trial in HIV-negative women (Gabon included), IPTp-MQ was no longer cost-effective compared with IPTp-SP when: the price of MQ was over US$ 0.87 per tablet; the price of SP was less than US$ 0.001 per tablet; the incidence of clinical malaria was less than 0.12 episodes per person-year at risk and the RR was over 0.81. Modest shifts of all the variables were allowed for IPTp-MQ to remain cost-effective with the exclusion of Gabon. For the trial in HIV-positive women, IPTp-MQ compared with IPTp-placebo was no longer cost-effective when the MQ price per tablet was higher than US$ 10.7 per tablet.

## Discussion

This economic evaluation found that at an estimated price of 0.50 US$ per tablet the addition of an effective antimalarial as IPTp is highly cost-effective in HIV-positive pregnant women receiving daily CTXp and in the context of LLINs. Moreover, this addition would bring modest household cost savings (on average about 2 US$ per pregnant woman) and would remain cost-effective up to a price of over 10 US$ per tablet of the antimalarial drug. In HIV-negative pregnant women a more effective antimalarial drug than SP may be considered as a cost-effective alternative for IPTp at the price of 0.50 US$ per tablet and up to US$ 0.80 per tablet.

IPTp with MQ was found highly cost-effective in HIV-positive women because of the large number of DALYs averted as a consequence of reducing non-obstetric admissions. In this trial in HIV-positive women, the reduction in non-obstetric admissions, mainly caused by infections, was consistent with a decrease in the incidence of morbidity outcomes both malaria-related and overall [51–54]. Moreover, malaria is known to cause immunosuppression, leading to increased risk and severity of other infections [55, 56]. As it has been shown in children in previous antimalarial drug trials, it is plausible that the decreased risk of malaria infection in the women who received MQ had an impact in reducing the incidence and/or severity of other infections [57].

Among HIV-negative pregnant women although the absolute differences between intervention groups in the outcomes were small (episodes person/year 0.17 and 0.12 in the SP and MQ group, respectively), IPTp with MQ was cost-effective mainly due to the economic impact on the reduction in the incidence of clinical malaria episodes and, therefore, on the health system costs of care seeking among pregnant women.

Although the two clinical trials this study is based on, have discarded MQ at the dose of 15mg/kg as the drug suitable for substituting SP or to integrating CTXp, they have shown the health benefits of using a more efficacious antimalarial than SP for HIV-negative, and of adding an efficacious antimalarial to daily CTXp for HIV-positive pregnant women. These findings were considered as notable and the cost-effectiveness analysis of an alternative antimalarial was modelled taking findings on MQ as an example. Importantly, while the need of an alternative antimalarial to SP for IPTp is very much recognized among the public health community, MQ is, up to now, the only one potential alternative option.

The choice of the outcomes used in this economic analysis was based on their public health impact as well as the statistically significant difference found between intervention groups. Intermediate health outcomes are generally used in economic evaluations when final health ones have not been measured [58]. However, final health outcomes are preferred when available from the study [25, 59]. In this case, although malaria parasitemia and placental infection were significantly reduced in the IPTp-MQ group, the use of a clinical outcome such as hospital admissions avoids making an hypothetical extrapolation of the parasitological outcome to one of public health relevance, leading to more direct and therefore, realistic estimates [29].

In the trial in HIV-negative women, the ICER resulting from the exclusion of Gabon was nearly twice the one resulting from its inclusion. Since the same drug costs were used across all settings, costs were largely driven by the higher cost of health personnel and other health service costs in Gabon, which led to higher health system savings arising from the reduction in the number of malaria cases. In practice, different prices may be applied in different countries due to market segmentation by pharmaceutical companies [60]; on the other hand, pooled procurement arrangements will tend to lead to price convergence. Assuming a common drug price, the implementation of a more efficacious malaria prevention tool in pregnancy will be more cost-effective, everything else constant, in settings where treatment cost is higher. However, from a public health perspective, the implementation of efficacious prevention is equally needed in any setting where the burden of disease is high and where it consumes a considerable share of health system resources [61].

This study differs from existing economic evaluations of IPTp in a number of aspects. First, we use a hypothetical price for MQ, since it is not currently procured in large quantities for public health programmes, while in previous economic evaluations of IPTp the price of the medicine was either known or not relevant to the study objectives [29, 62–67]. Second, the trial in HIV-negative women was the first economic evaluation comparing a different drug for IPTp instead of comparing the currently recommended IPTp-SP with “do-nothing”. Third, there are several differences between the current study and the only economic evaluation model that differentiated the impact of IPTp-SP by HIV status [64]. The latter mentioned model included both HIV-positive and negative pregnant women, while the current study estimates two separate models for the two groups of women. The health effect of IPTp-SP was represented by the reduction in placental parasitemia modeled to estimate the number of low birth weight cases averted, while the present study uses more direct health outcomes as in a previous study [29]. In addition, the previous study took place before the introduction of daily CTXp, while the current study estimates the cost-effectiveness of the introduction of an antimalarial for IPTp in addition to CTXp [68].

The choice of the values of parameters used to calculate DALYs has an impact on cost-effectiveness results [69]. In this study, country specific, instead of international life expectancies were used to provide local policy makers with useful information in their decision making process. However, results of this evaluation did not dramatically change even when the highest possible life expectancy level, the one of Japanese women, was used.

For HIV-positive women, the value of the loss of ability to work caused by the low tolerability to MQ in approximately 30% of trial participants, reduced indirect cost savings arising from the efficacy of the drug. For HIV-negative women, indirect cost savings were outweighed by the value of the reduced ability to work, translating savings into an indirect cost for the households. The economic value of low tolerability could be seen as a transfer of costs from the health system to pregnant women and ultimately to the whole community [70].

Safety concerns on MTCT of HIV were not taken into account in this economic evaluation for several reasons: they did not constitute study outcomes; they were found in an exploratory analysis of trial data; and the present study aimed at modelling the cost-effectiveness of an alternative drug for IPTp which will have to be safe, with no need, therefore, to consider such concerns.

In conclusion, adding IPTp with an effective antimalarial to daily CTXp in HIV-positive pregnant women is highly cost-effective. Moreover, IPTp with an efficacious alternative antimalarial is more cost-effective than IPTp-SP in HIV-negative pregnant women. However, the potential utility of MQ (15 mg/kg) for IPTp was compromised by low tolerability in both trials and the safety concerns in HIV-positive women. Therefore, further research is needed to find an alternative antimalarial to eventually replace SP for the prevention of malaria in HIV-negative pregnant women and to adequately protect HIV-positive pregnant women. In the latter case, given the potential impact that an effective antimalarial may have on the reduction of hospital admissions, the containment of its price should not be a priority.

## Supporting Information

S1 Fig Cumulative average of the net monetary benefits for IPTp-MQ versus IPTp-SP in HIV-negative pregnant women. IPTp-MQ = Intermittent Preventive Treatment of malaria in pregnancy with Mefloquine; IPTp-SP = Intermittent Preventive Treatment of malaria in pregnancy with Sulphadoxine-Phyrimethamine; HIV = Human Immunodeficiency Virus. This graph shows the cumulative average net monetary benefits (threshold level*incremental effects—incremental costs) with respect to the number of iterations performed in Monte Carlo simulations and allows to judge the number of iterations needed to produce stable results.
(TIF)

S2 Fig Cumulative average of the net monetary benefit for IPTp-MQ versus IPTp-Placebo in HIV-positive pregnant women. IPTp-MQ = Intermittent Preventive Treatment of malaria in pregnancy with Mefloquine; HIV = Human Immunodeficiency Virus. This graph shows the cumulative average net monetary benefits (threshold level*incremental effects—incremental costs) with respect to the number of iterations performed in Monte Carlo simulations and allows to judge the number of iterations needed to produce stable results.
(TIF)

S1 Table Characteristics of trials sites. ^1^In children; ^2^Quintuple mutations in pregnant women, data from 2011; ^3^Quintuple mutations prevalence in pregnant women who received placebo-IPTp in a RCT conducted in 2003–06; ^4^ In children, triple mutant prevalence in 2001; ^5^Ministry of Health- Republic of Kenya (2012),"Kenya, County HIV Service Delivery Profiles." National AIDS and STI Control Program; ^6^Gonzalez R. et al.(2012) HIV Med; ^7^Ministry of Health and Social Welfare-The United Republic of Tanzania (2008). "Surveillance of HIV and Syphilis Infections Among Antenatal Clinic Attendees." http://pmtct.or.tz/pmtct-tanzania/pmtct-in-tanzania/ [accessed February 2014]; ^8^ Ministry of Health- Republic of Kenya (2012). "Early Infant Diagnosis Program. National AIDS and STI Control Program." www.nascop.org/eid [accessed February 2014]; ^9^ Moraleda C. et al. (2014) J Acquir Immune Defic Syndr; ^10^Ministry of Health and Social Welfare-The United Republic of Tanzania (2013). "UNAIDS 2013 Global Report." http://pmtct.or.tz/pmtct-tanzania/pmtct-in-tanzania/ [accessed February 2014].
(DOCX)

S2 Table Trial in HIV-negative pregnant women: malaria related outcomes at delivery, findings from ITT^a^ analysis. ^a^ Intention to treat; ^b^ Low birth weight < 2500 gr; ^c^ Proportional difference; ^d^ Arithmetic difference
(DOCX)

S3 Table Trial in HIV-positive pregnant women: malaria related outcomes at delivery, findings from ITT^a^ analysis. ^a^ Intention to Treat (ITT) analysis adjusted by country.; ^b^ Risk ratio; ^c^ Mean difference; ^d^ Assessed by the Ballard score (excluding incomplete data).
(DOCX)

S4 Table Trial in HIV-negative pregnant women: Incidence of clinical malaria, outpatient visits and hospital admissions, findings from ITT^a^ analysis. ^a^ Intention to treat; ^b^ Episodes per person/year, adjusted by country.
(DOCX)

S5 Table Trial in HIV-positive pregnant women: Incidence of clinical malaria, outpatient visits and hospital admissions, findings from ITT^a^ analysis. ^a^ Intention to treat; ^b^ Episodes per person/year, adjusted by country.
(DOCX)
