# Supplementary figures and images for: Economic Evaluation of an Alternative Drug to Sulfadoxine-Pyrimethamine as Intermittent Preventive Treatment of Malaria in Pregnancy
Source: PLoS One. 2015 Apr 27;10(4):e0125072. doi: 10.1371/journal.pone.0125072 (PMC4410941; doi:10.1371/journal.pone.0125072)

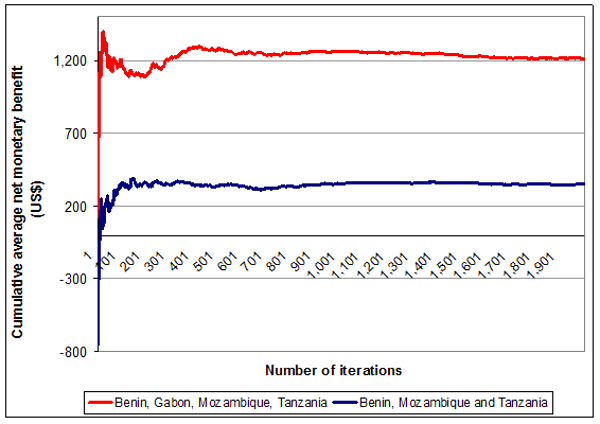

Supplement: S1 Fig — IPTp-MQ = Intermittent Preventive Treatment of malaria in pregnancy with Mefloquine; IPTp-SP = Intermittent Preventive Treatment of malaria in pregnancy with Sulphadoxine-Phyrimethamine; HIV = Human Immunodeficiency Virus. This graph shows the cumulative average net monetary benefits (threshold level*incremental effects—incremental costs) with respect to the number of iterations performed in Monte Carlo simulations and allows to judge the number of iterations needed to produce stable results. (TIF) [file pone.0125072.s001.tif]

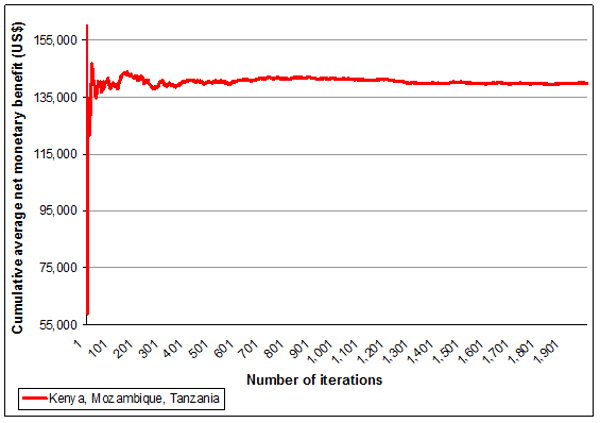

Supplement: S2 Fig — IPTp-MQ = Intermittent Preventive Treatment of malaria in pregnancy with Mefloquine; HIV = Human Immunodeficiency Virus. This graph shows the cumulative average net monetary benefits (threshold level*incremental effects—incremental costs) with respect to the number of iterations performed in Monte Carlo simulations and allows to judge the number of iterations needed to produce stable results. (TIF) [file pone.0125072.s002.tif]
