# Supplementary material for: Economic Evaluation of an Alternative Drug to Sulfadoxine-Pyrimethamine as Intermittent Preventive Treatment of Malaria in Pregnancy
Source: PLoS One. 2015 Apr 27;10(4):e0125072. doi: 10.1371/journal.pone.0125072 (PMC4410941; doi:10.1371/journal.pone.0125072)
Supplement: S1 Table — 1In children; 2Quintuple mutations in pregnant women, data from 2011; 3Quintuple mutations prevalence in pregnant women who received placebo-IPTp in a RCT conducted in 2003–06; 4 In children, triple mutant prevalence in 2001; 5Ministry of Health- Republic of Kenya (2012),"Kenya, County HIV Service Delivery Profiles." National AIDS and STI Control Program; 6Gonzalez R. et al.(2012) HIV Med; 7Ministry of Health and Social Welfare-The United Republic of Tanzania (2008). "Surveillance of HIV and Syphilis Infections Among Antenatal Clinic Attendees." http://pmtct.or.tz/pmtct-tanzania/pmtct-in-tanzania/ [accessed February 2014]; 8 Ministry of Health- Republic of Kenya (2012). "Early Infant Diagnosis Program. National AIDS and STI Control Program." www.nascop.org/eid [accessed February 2014]; 9 Moraleda C. et al. (2014) J Acquir Immune Defic Syndr; 10Ministry of Health and Social Welfare-The United Republic of Tanzania (2013). "UNAIDS 2013 Global Report." http://pmtct.or.tz/pmtct-tanzania/pmtct-in-tanzania/ [accessed February 2014]. (DOCX) [file pone.0125072.s003.docx]

| **Country** | **Benin** | **Gabon** | **Kenya** | **Mozambique** | **Tanzania** |
| --- | --- | --- | --- | --- | --- |
| **Trial** | - HIV-negative pregnant women | - HIV-negative pregnant women | - HIV-positive pregnant women | - HIV-negative pregnant women - HIV-positive pregnant women | - HIV-negative pregnant women - HIV-positive pregnant women |
| **Sites** | - Allada - Sékou - Attogon | - Lambaréné - Fougamou | - Siaya | - Manhiça - Maragra | - Makole - Chamwino |
| **Malaria Transmission** | Hyperendemic | Hyperendemic | Holoendemic | Mesoendemic | Mesoendemic |
| **High season** | Apr-Jul | Oct-May | May-Jul | Sep-Mar | Jun-Aug |
| ***P. falciparum* infection** | >90% | >90% | >90% | >90% | >90% |
| **SP resistance markers** | >75%^1^ | >80%^2^ | - | 12%^3^ | <25%^4^ |
| **HIV prevalence in pregnant women** | - | - | 18.4%^5^ | 29%^6^ | 6.9%^7^ |
| **Perinatal MTCT of HIV** | - | - | 6%^8^ | 9%^9^ | 15%^10^ |
