# Supplementary material for: Economic Evaluation of an Alternative Drug to Sulfadoxine-Pyrimethamine as Intermittent Preventive Treatment of Malaria in Pregnancy
Source: PLoS One. 2015 Apr 27;10(4):e0125072. doi: 10.1371/journal.pone.0125072 (PMC4410941; doi:10.1371/journal.pone.0125072)
Supplement: S2 Table — a Intention to treat; b Low birth weight < 2500 gr; c Proportional difference; d Arithmetic difference (DOCX) [file pone.0125072.s004.docx]

|  | **Sulfadoxine-pyrimethamine** | | **Mefloquine** | | **RR or Difference** | **95% CI** | **p-value** |
| --- | --- | --- | --- | --- | --- | --- | --- |
|  | **n/N** | **%** | **n/N** | **%** |  |  |  |
| ***Primary endpoint:*** | | | | | | | |
| Overall prevalence of Low Birth Weight^b^ | 177/1398 | 12.7 | 360/2778 | 13.0 | 1.02 | (0.86; 1.22) | 0.8 |
| ***Secondary endpoints:*** | | | | | | | |
| Maternal parasitemia by observed microscopy (OM) | 63/1372 | 4.6 | 88/2737 | 3.2 | 0.70 | (0.51; 0.96) | 0.03 |
| Parasitemia density (slides positive by OM), geometric mean [IQR] | 3141 [1032; 11763] | | 4689 [1049; 35902] | | 1.51^c^ | (0.67; 3.40) | 0.32 |
| Placental infection (Histology or smear) | 72/1281 | 5.6 | 119/2568 | 4.6 | 0.83 | (0.63; 1.10) | 0.19 |
| Maternal anaemia at delivery (Hb<11 g/dl) | 609/1380 | 44.1 | 1110/2743 | 40.5 | 0.92 | (0.85; 0.99) | 0.03 |
| Severe maternal anemia at delivery (Hb<7 g/dl) | 15/1380 | 1.1 | 15/2743 | 0.5 | 0.5 | (0.25; 1.02) | 0.06 |
| Maternal Hb, mean (SD)[n] | 11.0 (1.6) [1380] | | 11.1 (1.5) [2743] | | 0.15^d^ | (0.05; 0.25) | 0.003 |
| Cord blood parasitemia by OM | 4/1337 | 0.3 | 6/2667 | 0.2 | 0.74 | (0.21 ;2.62) | 0.64 |
| Cord blood anemia (Hb < 12.5g/dl) | 170/1334 | 12.7 | 353/2672 | 13.2 | 1.03 | (0.87; 1.22) | 0.71 |
| Maternal parasitemia by OM one month after delivery | 21/1149 | 1.8 | 42/2281 | 1.8 | 1.01 | (0.60; 1.69) | 0.98 |
