# Supplementary material for: Economic Evaluation of an Alternative Drug to Sulfadoxine-Pyrimethamine as Intermittent Preventive Treatment of Malaria in Pregnancy
Source: PLoS One. 2015 Apr 27;10(4):e0125072. doi: 10.1371/journal.pone.0125072 (PMC4410941; doi:10.1371/journal.pone.0125072)
Supplement: S3 Table — a Intention to Treat (ITT) analysis adjusted by country.; b Risk ratio; c Mean difference; d Assessed by the Ballard score (excluding incomplete data). (DOCX) [file pone.0125072.s005.docx]

|  | **Placebo** | | | **Mefloquine** | | | **RR or Difference** | **95% CI** | **p-value** |
| --- | --- | --- | --- | --- | --- | --- | --- | --- | --- |
|  | **n/N** | **%** | | **n/N** | **%** | |  |  |  |
| ***Primary endpoint:*** | | | | | | | | | |
| Maternal parasitemia (smear or PCR) | 37/490 | | 7.6 | 17/483 | | 3.5 | 0.47^b^ | (0.27; 0.82) | 0.008 |
| ***Secondary endpoints:*** | | | | | | | | | |
| Placental infection (histology, smear or PCR) | 34/462 | | 7.4 | 17/449 | | 3.8 | 0.52^b^ | (0.29; 0.90) | 0.021 |
| Maternal anemia (Hb<11 g/dl) | 187/484 | | 38.6 | 190/479 | | 39.7 | 1.02^b^ | (0.88; 1.19) | 0.758 |
| Severe maternal anemia (Hb <7g/dl) | 12/484 | | 2.5 | 11/479 | | 2.3 | 0.93^b^ | (0.41; 2.08) | 0.857 |
| Maternal Hb, mean (SD)[n] | 11.3 (2.2) [484] | | | 11.2 (2.1) [479] | | | -0.03^c^ | (-0.28; 0.22) | 0.826 |
| Low birth weight (< 2500g) | 46/486 | | 9.5 | 61/489 | | 12.5 | 1.32^b^ | (0.90; 1.95) | 0.157 |
| Gestational age at birth (weeks), mean (SD) [n]^d^ | 38.8 (1.1) [230] | | | 38.7 (1.3) [236] | | | -0.10^c^ | (-0.32; 0.13) | 0.405 |
| Cord blood parasitemia (smear) | 3/462 | | 0.6 | 1/471 | | 0.2 | 0.33^b^ | (0.03; 3.13) | 0.334 |
| Cord blood anemia (Hb <12.5 g/dl) | 67/459 | | 14.6 | 80/471 | | 17 | 1.17^b^ | (0.87; 1.58) | 0.303 |
| Maternal parasitemia one month post- delivery (smear) | 7/423 | | 1.7 | 8/413 | | 1.9 | 1.20^b^ | (0.44; 3.26) | 0.721 |
