# Supplementary material for: Economic Evaluation of an Alternative Drug to Sulfadoxine-Pyrimethamine as Intermittent Preventive Treatment of Malaria in Pregnancy
Source: PLoS One. 2015 Apr 27;10(4):e0125072. doi: 10.1371/journal.pone.0125072 (PMC4410941; doi:10.1371/journal.pone.0125072)
Supplement: S4 Table — a Intention to treat; b Episodes per person/year, adjusted by country. (DOCX) [file pone.0125072.s006.docx]

|  | **SP** | | **MQ** | |  |  |  |
| --- | --- | --- | --- | --- | --- | --- | --- |
|  | **N/PYAR^b^** | **Incidence^b^** | **N/PYAR** | **Incidence** | **Relative Rate** | **95% CI** | **p-value** |
| ***Secondary endpoints***: |  |  |  |  |  |  |  |
| Clinical malaria | 96/551.8 | 0.17 | 130/1103.2 | 0.12 | 0.67 | (0.52; 0.88) | 0.004 |
| Outpatients visits | 850/557.8 | 1.52 | 1480/1110.1 | 1.33 | 0.86 | (0.78; 0.95) | 0.003 |
| All cause hospital admissions | 106/557.8 | 0.19 | 186/1110.1 | 0.17 | 0.88 | (0.68; 1.14) | 0.35 |
| Non-obstetric hospital admission | 91/557.8 | 0.16 | 166/1110.1 | 0.15 | 0.92 | (0.69;1.22) | 0.56 |
