# Supplementary material for: Economic Evaluation of an Alternative Drug to Sulfadoxine-Pyrimethamine as Intermittent Preventive Treatment of Malaria in Pregnancy
Source: PLoS One. 2015 Apr 27;10(4):e0125072. doi: 10.1371/journal.pone.0125072 (PMC4410941; doi:10.1371/journal.pone.0125072)
Supplement: S5 Table — a Intention to treat; b Episodes per person/year, adjusted by country. (DOCX) [file pone.0125072.s007.docx]

|  | **Control** | | **Mefloquine** | | **Relative Rate** | **95% CI** | **p-value** |
| --- | --- | --- | --- | --- | --- | --- | --- |
|  | **N/PYAR^b^** | **Incidence** | **N/PYAR^b^** | **Incidence** |  |  |  |
| ***Secondary outcomes:*** |  |  |  |  |  |  |  |
| Clinical malaria | 16/189.1 | 0.09 | 8/182.2 | 0.04 | 0.52 | (0.22; 1.21) | 0.128 |
| Outpatient visits | 401/190.2 | 2.11 | 332/182.8 | 1.82 | 0.86 | (0.72; 1.03) | 0.098 |
| All-cause hospital admissions | 68/190.2 | 0.36 | 41/182.8 | 0.22 | 0.65 | (0.41; 1.03) | 0.065 |
| Non-obstetric hospital admissions | 67/190.2 | 0.35 | 37/182.8 | 0.2 | 0.59 | (0.37; 0.95) | 0.031 |
